# Efficacy of whole-brain radiotherapy with or without simultaneous integrated boost in non-small cell lung cancer with brain metastases: a retrospective analysis

**DOI:** 10.3389/fmed.2025.1733289

**Published:** 2026-01-12

**Authors:** Bingxin Zhao, Meng Zhang, Wenqian Fu, Lan Wang, Wen Gao, Tianhui Guo, Haiji Wang, Biyuan Zhang, Qi Wang

**Affiliations:** Department of Radiation Oncology, The Affiliated Hospital of Qingdao University, Qingdao, Shandong, China

**Keywords:** brain metastases, non-small cell lung cancer, propensity score matching, simultaneous integrated boost, whole brain radiotherapy

## Abstract

**Background:**

Whole-brain radiotherapy (WBRT) remains a cornerstone in the management of brain metastases (BMs) from non-small cell lung cancer (NSCLC), and WBRT with simultaneous integrated boost (WBRT-SIB) emerges as a promising strategy that aims to improve local tumor control through dose escalation while maintaining the coverage of subclinical disease offered by WBRT in theory. However, current evidence regarding the efficacy of WBRT-SIB remains inconclusive. This study aimed to compare the intracranial efficacy of WBRT and WBRT-SIB in NSCLC patients with BMs.

**Methods:**

Clinical data from 119 patients with NSCLC-BM treated between 2019 and 2025 were retrospectively analyzed. Local progression-free survival (local PFS) was the primary endpoint, while distant PFS and intracranial PFS (iPFS) were secondary endpoints. Propensity score matching (PSM) was performed to balance baseline characteristics. Kaplan–Meier and Cox regression analyses were applied to identify prognostic factors.

**Results:**

No significant differences were observed in local PFS, distant PFS, or iPFS between the WBRT and WBRT-SIB groups, both before and after PSM (all *p* > 0.05). Subgroup analysis revealed that patients with fewer than eight BMs who received WBRT-SIB achieved significantly longer local PFS compared with those treated with WBRT (*p* = 0.043), along with a trend toward improved iPFS that did not reach statistical significance (*p* = 0.066). Furthermore, anti-angiogenic therapy showed a trend as a protective factor for local PFS without reaching statistical significance (*p* = 0.086).

**Conclusion:**

WBRT-SIB provided comparable overall intracranial control to conventional WBRT but achieved superior local tumor control in patients with limited brain metastases (< 8 lesions). These findings support WBRT-SIB as a promising option for selected NSCLC-BM patients, warranting validation in prospective studies.

## Introduction

Brain metastases (BMs) are the most common type of intracranial tumors, occurring in up to one-third of patients with systemic cancers (1). The increasing incidence of BMs originating from non-small cell lung cancer (NSCLC) has become a significant clinical concern. In NSCLC patients, over 10% present with BMs at the time of initial diagnosis, and among those with metastatic disease, the incidence exceeds 20% (2). Consequently, the management of BMs in NSCLC has remained a central focus of ongoing clinical research.

Conventional chemotherapy has shown limited efficacy in treating BMs, primarily due to its inability to penetrate the blood–brain barrier. As a result, whole-brain radiotherapy (WBRT) has remained the mainstay of treatment for several decades (3, 4). The pursuit of improved local control, on the other hand, has driven the adoption of more targeted approaches such as stereotactic radiosurgery (SRS) for selected patients, especially those with good performance status and limited intracranial disease. Multiple randomized clinical trials have demonstrated that patients with a limited number of BMs who receive an SRS boost achieve improved local control (5). Nevertheless, a substantial proportion of patients are ineligible for SRS due to factors such as high lesion burden, increased risk of intracranial recurrence compared to WBRT, lack of access to SRS technology, or other comorbid conditions (6).

Recent advancements in conformal radiation techniques, particularly intensity-modulated radiation therapy (IMRT), have led to the development of novel strategies that aim to integrate the benefits of both WBRT and SRS (7, 8). IMRT enables the precise delivery of differential radiation doses to tumor sites while sparing surrounding normal tissues. This has facilitated the emergence of WBRT with simultaneous integrated boost (WBRT-SIB), a technique that delivers higher doses directly to metastatic lesions while maintaining a standard whole-brain dose. In theory, this approach targets subclinical disease and may reduce recurrence risk (9–12). However, it remains uncertain whether the addition of a localized boost translates into a definitive improvement in tumor control, and consensus among institutions is still lacking. Moreover, most existing studies have not limited patient populations by tumor type or included patients with high BM burden, thereby limiting their applicability to real-world clinical scenarios. Further research is needed to clarify the optimal indications, efficacy, and recurrence patterns associated with WBRT and WBRT-SIB.

The objective of this study was to evaluate the effectiveness of WBRT compared to WBRT-SIB in the treatment of BMs from NSCLC. Additionally, it aimed to investigate the common patterns of intracranial progression. The findings are intended to identify the most effective therapeutic approach and contribute to the development of personalized treatment strategies.

## Materials and methods

### Patients

Clinical data from NSCLC-BM patients who underwent WBRT or WBRT-SIB at the Affiliated Hospital of Qingdao University were retrospectively collected between October 2019 and June 2025. This study was approved by the ethics committees of the Affiliated Hospital of Qingdao University, China. Given the retrospective nature of the study, the requirement for informed consent was waived by the ethics committees.

The inclusion criteria were as follows: (1) patients with a confirmed pathological diagnosis of NSCLC; (2) BMs confirmed by contrast-enhanced magnetic resonance imaging (MRI) within 1 month prior to radiotherapy; (3) use of radiotherapy with WBRT or WBRT-SIB, and (4) availability of necessary clinical data. The exclusion criteria included: (1) patients who had received other local treatments, such as tumor resection or stereotactic radiotherapy (SRT); (2) absence of follow-up data; (3) missing imaging data; (4) patients with leptomeningeal disease; and (5) a follow-up period of less than 3 months. The exclusion of SRT patients was necessitated by substantial baseline differences (particularly in tumor burden) compared to the WBRT/WBRT-SIB groups in our center, which would have confounded a comparative analysis, despite SRT being a standard treatment for limited BMs. Leptomeningeal disease was assessed and ruled out based on clinical evaluation and contrast-enhanced brain MRI. Ultimately, 119 patients were included in this study.

Baseline characteristics were collected, including age, sex, the number of BMs, sum of the longest diameters of BMs, total volume of BMs, the graded prognostic assessment (GPA), clinicopathological type, extracranial metastatic status, concurrent systemic treatment, and driver gene status, which refers to the presence of genetic alterations in genes known to drive oncogenesis and guide the selection of targeted therapies, including but not limited to epidermal growth factor receptor (EGFR), anaplastic lymphoma kinase (ALK), ROS proto-oncogene 1 (ROS1), Kirsten rat sarcoma viral oncogene homolog (KRAS), B-Raf proto-oncogene (BRAF), human epidermal growth factor receptor 2 (HER2/ERBB2), and RET proto-oncogene (RET). The sum of the longest diameters of BMs was calculated by manually reviewing the imaging slices, measuring each lesion, and summing the longest diameters of all BMs identified solely on the baseline contrast-enhanced MRI scan. The GPA score was determined using the NSCLC-specific indices via the online calculator.^1^ Concurrent systemic treatment was defined as any systemic anti-cancer therapy (including chemotherapy, immunotherapy, targeted therapy, or anti-angiogenic therapy) administered within a period spanning from 1 month before the start of radiotherapy to 1 month after its completion. Additionally, the start and end dates of radiotherapy and the date and pattern of intracranial progression were recorded.

### Radiotherapy strategy

Radiotherapy was administered using either WBRT or WBRT-SIB, and the radiation treatment plan was discussed and decided by two radiation oncologists with over 20 years of experience, based on factors such as the overall burden of BMs, lesion size and distribution, the patient’s general condition, and prognosis. Patients were positioned in the supine position, and a thermoplastic mask was used to ensure head immobilization. Enhanced computed tomography (CT) scans were performed to precisely localize the treatment area, extending from the cranial vault to the cricoid cartilage, with a slice thickness of 2 mm. These imaging data were then integrated into the ECLIPSE planning system and merged with dynamic contrast-enhanced MRI of the brain. The target volumes were delineated within the ECLIPSE system, with the gross tumor volume (GTV) defined as the visible metastatic lesions on the imaging, and the clinical target volume (CTV) encompassing the entire brain. Both the GTV and CTV were expanded by 2 mm to define the planning gross tumor volume (P-GTV) and planning clinical target volume (P-CTV), respectively.

Additionally, critical organs at risk, including the optic nerve, optic chiasm, eyes, lenses and brainstem, were outlined. The radiotherapy regimen was tailored to meet individual patient needs. The majority of patients received a total dose to the planning target volume (PTV) of either 30 Gy in 10 fractions or 40 Gy in 20 fractions. The dose administered to the P-GTV (i.e., the simultaneous integrated boost) was adjusted based on the maximum diameter and location of the brain metastasis.

### Follow-up and endpoint

Data were collected from the medical records of hospitalized patients. Clinical efficacy was assessed, with the final results derived from the data obtained during the most recent follow-up visit. The primary endpoint of the study was local progression-free survival (local PFS), while the secondary endpoints included distant progression-free survival (distant PFS) and intracranial progression-free survival (iPFS). Progression was defined as a > 20% increase in BM diameter or the emergence of new BMs, as identified through contrast-enhanced brain MRI, in accordance with the RECIST 1.1 guidelines.

Local PFS was defined as the time from the completion of radiotherapy to the first occurrence of a > 20% increase in the diameter of pre-existing BMs. Distant PFS was defined as the time from the cessation of radiotherapy to the emergence of new BMs, while iPFS was measured from the end of radiotherapy to the occurrence of intracranial progression. Patients who died without documented local progression, or who were lost to follow-up, were censored at the date of death or last follow-up. Imaging assessments using contrast-enhanced brain MRI were systematically reviewed at 3-month intervals following the completion of radiotherapy. All endpoints were censored at the final follow-up (data cutoff: June 2025) if no respective events were observed.

### Statistical analysis

In this study, the analysis was performed using R Studio (version 4.5.1). For continuous variables, statistical comparisons between two groups were performed using the Welch’s *t*-test when data followed a normal distribution but violated the assumption of homogeneity of variances. The Wilcoxon rank-sum test was employed when the normality assumption was not met. The chi-square test was used to assess differences between the two groups regarding categorical variables. To manage the unbalance of potential interference factors, propensity score matching (PSM) was adopted to set up two treatment groups with an even distribution of original characteristics. The PSM analysis was used between WBRT and WBRT-SIB group to control confounding factors of patients, and performed with a logistic regression that considered the following factors: age, sex, GPA, the number and the sum of the longest diameters of BMs, histological status, driver gene status, intracranial symptoms, the existing of extracranial metastases and concurrent systemic treatments. The Kaplan-Meier method was employed to evaluate local PFS, distant PFS, and iPFS for both groups, and survival curves were plotted. Additionally, Cox regression analysis was conducted for various evaluations. Both univariate and multivariate analyses utilized Cox proportional hazards regression models to identify independent prognostic factors. After the univariate analysis, clinical factors with a significance level of *p* < 0.05 were included in the multivariate Cox proportional hazards regression model. The results were reported as hazard ratios (HR) with 95% confidence intervals (CI). All statistical tests were two-tailed, with a *P*-value threshold of < 0.05 considered statistically significant.

## Results

### Baseline characteristics

**FIGURE 1:**
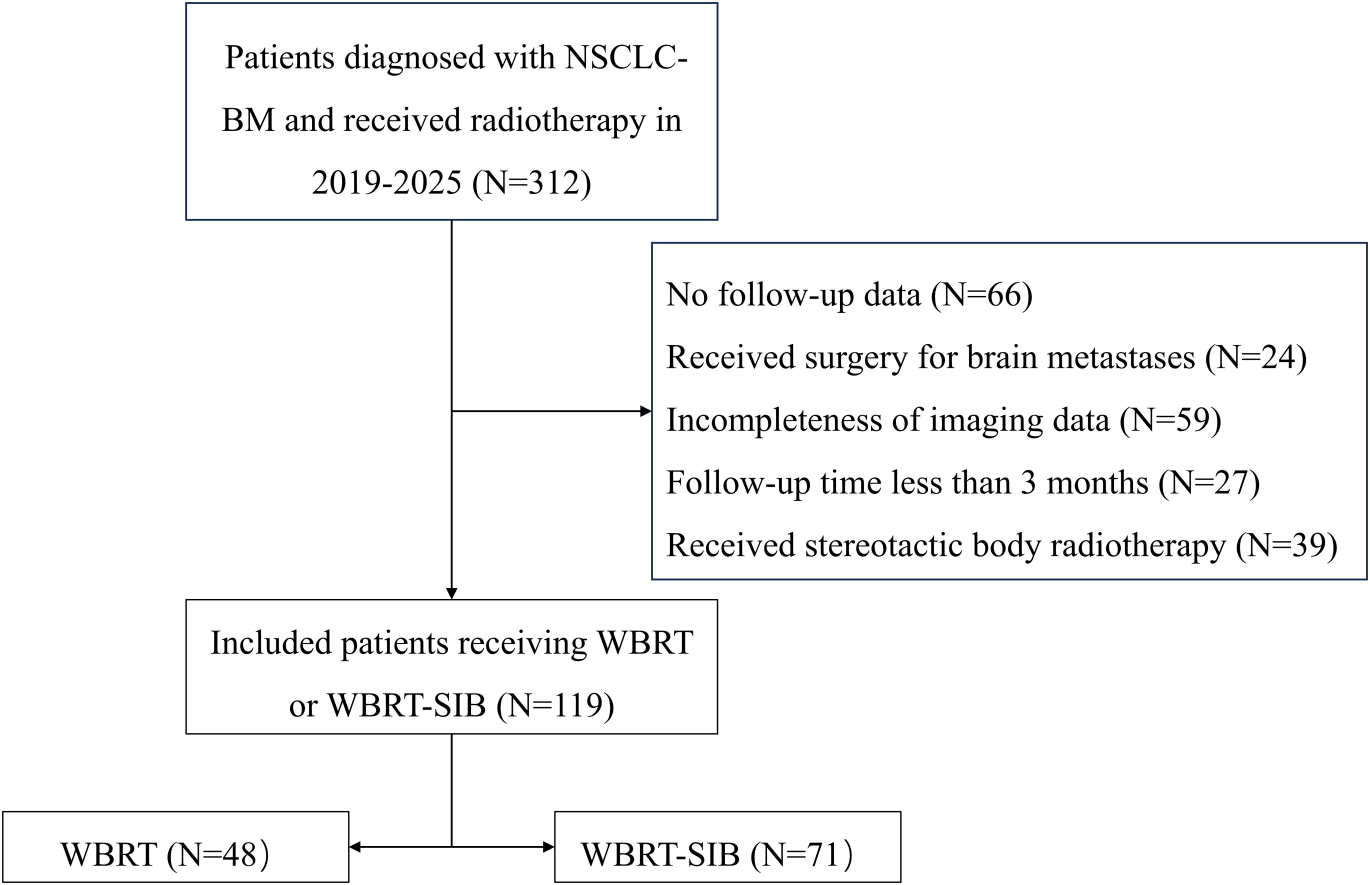
The flowchart of the database filtering process.

This study included a total of 312 individuals diagnosed with NSCLC-BM who received radiotherapy at the Affiliated Hospital of Qingdao University between October 2019 and June 2025. Among them, 119 patients were selected for inclusion, consisting of 48 individuals who underwent WBRT and 71 who received WBRT-SIB (Figure 1). The overall characteristics of the patient cohort are provided in Table 1. Specifically, patients who underwent WBRT presented with a greater number of BMs and a larger sum of maximum diameter (*p* < 0.001 and *p* = 0.005, respectively). Moreover, the WBRT cohort demonstrated a higher proportion of patients harboring driver gene alterations and receiving target therapy (*p* = 0.003 and *p* = 0.002, respectively). Age, sex, GPA, total volume of BMs, histological status, neurologic symptoms, extracranial metastatic status, chemotherapy, anti-angiogenic therapy, and immunotherapy were comparably distributed across both groups.

**TABLE 1:** Patient characteristics.

Characteristics	WBRT	WBRT-SIB	*P*-value
*n*	48	71	
Age, median (IQR)	62 (57.5, 67)	61 (56, 68)	0.916
Sex, n (%)			0.232
Male	28 (58.3%)	49 (69.0%)	
Female	20 (41.7%)	22 (31.0%)	
GPA, median (IQR)	2 (1.5, 2.5)	2 (2, 2.5)	0.381
Number of BMs, median (IQR)	9 (5.75, 30.25)	4 (2, 11)	< 0.001
Sum of maximum diameter of BMs, median (IQR)	9.295 (4.38, 25.85)	5.02 (2.77, 10.235)	0.005
Total volume of BMs, median (IQR)	15.344 (9.7051, 91.722)	36.598 (7.3632, 98.64)	0.805
Histological status, n (%)			0.332
Large cell carcinoma	4 (8.3%)	7 (9.9%)	
Adenocarcinoma	44 (91.7%)	61 (85.9%)	
Squamous cell	0 (0%)	3 (4.2%)	
Driver gene status, n (%)			0.003
Negative	10 (20.8%)	34 (47.9%)	
Positive	38 (79.2%)	37 (53.1%)	
Neurologic symptoms, n (%)			0.232
Yes	28 (58.3%)	49 (69.0%)	
None	20 (41.7%)	22 (31.0%)	
Extracranial metastases, n (%)			0.597
Yes	24 (50.0%)	32 (45.1%)	
None	24 (50.0%)	39 (54.9%)	
Chemotherapy, n (%)			0.565
Yes	30 (62.5%)	48 (67.6%)	
None	18 (37.5%)	23 (32.4%)	
Target therapy, n (%)			0.002
Yes	36 (75.0%)	33 (46.5%)	
None	12 (25.0%)	38 (53.5%)	
Anti-angiogenic therapy, n (%)			0.502
Yes	22 (45.8%)	37 (52.1%)	
None	26 (54.2%)	34 (47.9%)	
Immunotherapy, n (%)			0.121
Yes	6 (12.5%)	17 (23.9%)	
None	42 (87.5%)	54 (76.1%)	

A detailed breakdown of therapeutically actionable driver alterations among the 75 driver gene-positive patients is provided. In the WBRT group (*n* = 38), EGFR mutations and ALK rearrangements were identified in 34 and 4 patients, respectively. In the WBRT-SIB group (*n* = 37), EGFR mutations and ALK rearrangements were present in 31 and 6 patients, respectively. No other actionable alterations, such as in ROS1, were detected in the cohort.

The median PTV dose was 40 Gy [interquartile range (IQR): 30–40 Gy] in the WBRT group and 40 Gy (IQR: 35–40 Gy) in the WBRT-SIB group, with no statistically significant difference between the two cohorts (*p* = 0.390). The median dose for the P-GTV boost was 54 Gy (IQR: 50–56 Gy), typically delivered in 2.3–5 Gy per fraction.

### Timing and rationale for radiotherapy relative to systemic therapy

Among the 119 enrolled patients, the majority (*n* = 94) proceeded to radiotherapy immediately following the MRI-based diagnosis of BMs, comprising 38 patients in the WBRT group and 56 in the WBRT-SIB group. Of these 94 patients, 58 were driver gene-positive. The rationale for initiating radiotherapy without first attempting or deferring to systemic therapy alone was: the presence of neurological symptoms (e.g., dizziness, headache, gait instability) at the time of BM diagnosis (*n* = 29), or the development of new BMs while already receiving targeted therapy for known systemic disease (*n* = 22). A small subset (*n* = 7) commenced radiotherapy before their driver gene test results were available and subsequently started targeted therapy within 1 month after completing radiation.

The remaining 25 patients were managed with initial systemic therapy upon BM diagnosis. They subsequently received radiotherapy only after intracranial progression was confirmed on follow-up MRI during systemic treatment (WBRT: *n* = 10; WBRT-SIB: *n* = 15). Prior to this progression, the systemic regimens in this subgroup were as follows: all 17 driver gene-positive patients had been on targeted therapy, while the 8 driver gene-negative patients received chemotherapy (*n* = 3) or immunotherapy (*n* = 5).

Critically, no patient in either group started a new line of targeted therapy following radiotherapy, thereby ensuring that the assessment of intracranial efficacy was not confounded after radiation.

### Prognostic information

**FIGURE 2:**
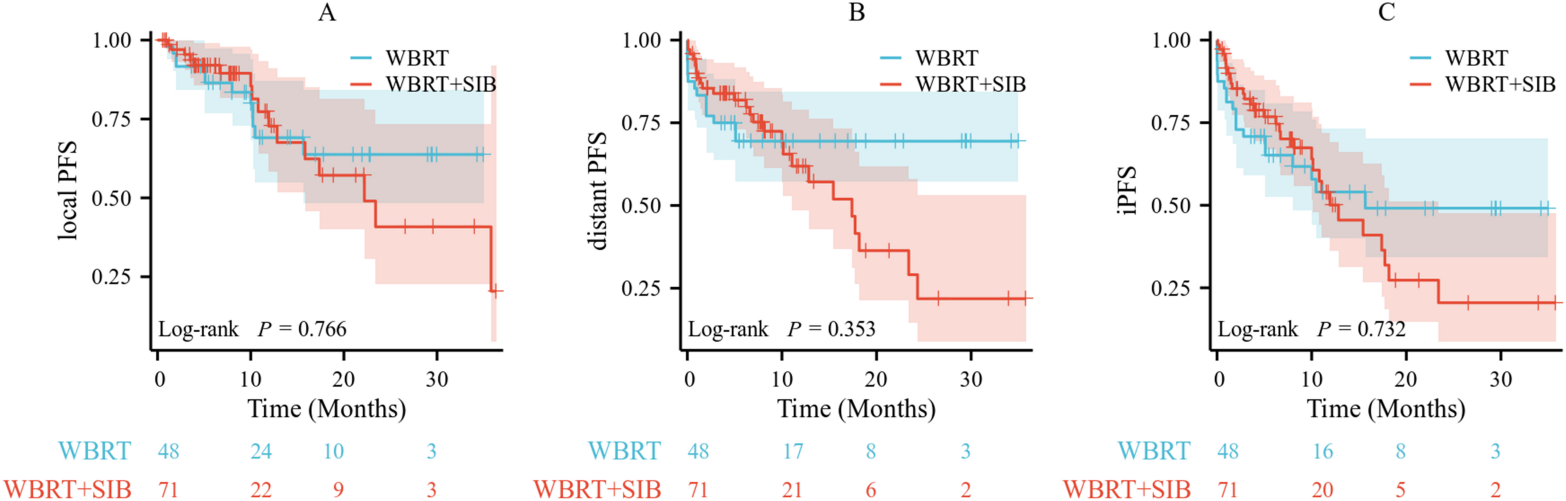
Comparison of local PFS **(A)**, distant PFS **(B)**, and iPFS **(C)** between WBRT group and WBRT-SIB group. Local PFS, local progression-free survival; distant PFS, distant progression-free survival; iPFS, intracranial progression-free survival; WBRT, whole brain radiotherapy; WBRT-SIB, whole brain radiotherapy with a simultaneous integrated boost.

The median follow-up period for the study cohort was 9.3 months, ranging from 3.2 to 52.0 months. The median local PFS was 7.9 months, the median distant PFS was 6.0 months, and the median iPFS was 5.4 months. At the time of the final follow-up, a total of 50 cases of intracranial progression were observed across both the WBRT and WBRT-SIB treatment groups. Among patients who received WBRT, 12 experienced local progression, and 14 developed new BMs. In contrast, among those treated with WBRT-SIB, 16 exhibited local progression, while 26 developed new BMs. Importantly, no statistically significant differences were found in local PFS, distant PFS, and iPFS between the WBRT and WBRT-SIB groups, with *p*-values of 0.766, 0.353, and 0.732, respectively (Figure 2).

The results of the univariate Cox regression analyses for local PFS are presented in Table 2. As no clinical factors reached the pre-specified significance level of *p* < 0.05 for inclusion, no multivariate model was constructed. However, it is noteworthy that anti-angiogenic therapy showed a trend as a protective factor for local PFS, although this association did not reach statistical significance (HR = 0.514, 95% CI 0.239–1.102, *p* = 0.086).

**TABLE 2:** Survival-related factors on local PFS in univariate analysis.

Characteristics	Total (N)	Hazard ratio (95% CI)	*P*-value
Radiotherapy	119		
WBRT	48	Reference	0.670
WBRT + SIB	71	1.181 (0.550–2.535)	
Ages	119	1.000 (0.960–1.043)	0.993
Sex	119		
Male	77	Reference	0.541
Female	42	1.277 (0.583–2.797)	
GPA	119	0.584 (0.313–1.092)	0.092
Number of BMs	119	0.999 (0.978–1.021)	0.949
Sum of maximum diameter of BMs	119	1.006 (0.997–1.015)	0.185
Sum of volume of BMs	119	0.997 (0.991–1.002)	0.231
Histological status	119		
Large cell carcinoma	11	Reference	0.998
Adenocarcinoma	105	1.001 (0.000–Inf)	
Squamous cell	3	1.002 (0.000–Inf)	1.000
Driver gene status	119		
Negative	44	Reference	0.769
Positive	75	0.889 (0.406–1.945)	
Neurologic symptoms	119		
Yes	77	Reference	0.224
None	42	0.864 (0.377–1.980)	
Extracranial metastases	119		
Yes	56	Reference	0.184
None	63	0.634 (0.296–1.359)	
Chemotherapy	119		
Yes	78	Reference	0.859
None	41	0.923 (0.424–2.006)	
Target therapy	119		
Yes	69	0.774 (0.368–1.625)	0.438
None	50	Reference	
Anti-angiogenic therapy	119		
Yes	59	0.514 (0.239–1.102)	0.086
None	60	Reference	
Immunotherapy	119		
Yes	23	Reference	0.887
None	96	0.925 (0.312–2.746)	

### PSM analysis

**FIGURE 3:**
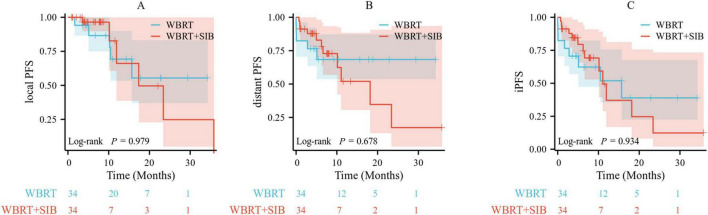
Comparison of local PFS **(A)**, distant PFS **(B)**, and iPFS **(C)** between the WBRT and WBRT-SIB groups in patients after PSM matching. Local PFS, local progression-free survival; distant PFS, distant progression-free survival; iPFS, intracranial progression-free survival; PSM, propensity score matching; WBRT, whole brain radiotherapy; WBRT-SIB, whole brain radiotherapy with a simultaneous integrated boost.

A 1:1 PSM was performed for all enrolled patients (48 in the WBRT group and 71 in the WBRT + SIB group), resulting in 34 matched patients in each group with well-balanced baseline characteristics. Further survival analyses were conducted in these 68 patients with BMs. The results indicated that, after PSM, there remained no significant differences in local PFS, distant PFS, or iPFS between the WBRT and WBRT + SIB groups (*p* = 0.979, 0.678, and 0.934, respectively) (Figure 3).

### Subgroup analysis

**FIGURE 4:**
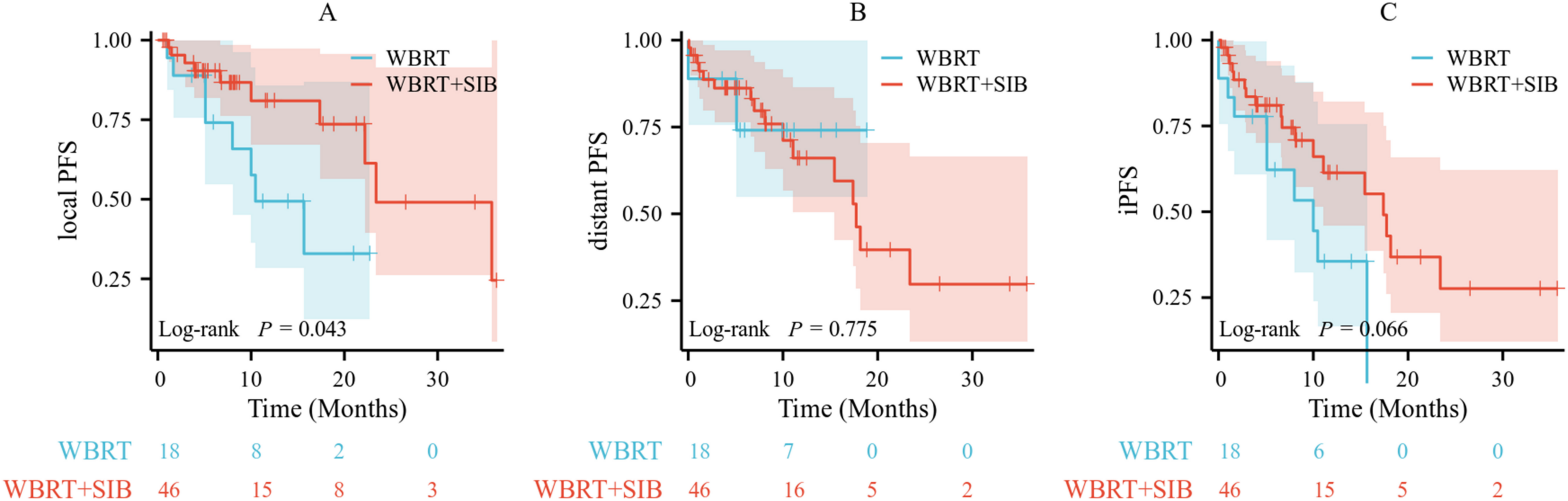
Comparison of local PFS **(A)**, distant PFS **(B)**, and iPFS **(C)** for WBRT group and WBRT-SIB group in patients with the number of BMs fewer than eight using Kaplan-Meier method. Local PFS, local progression-free survival; distant PFS, distant progression-free survival; iPFS, intracranial progression-free survival; WBRT, whole brain radiotherapy; WBRT-SIB, whole brain radiotherapy with a simultaneous integrated boost.

Due to the observed disparities in baseline characteristics between the WBRT and WBRT-SIB groups, a subgroup analysis was conducted. The cut-off of 8 BMs was adopted based on its use as an upper limit in a prior phase II trial (13). Among patients with fewer than 8 BMs (18 in the WBRT group and 46 in the WBRT-SIB group), the baseline characteristics of the two groups were further detailed in Supplementary Table S1. Notably, while the number of BMs, sum of maximum diameters, and volume were well-balanced between the treatment arms, significant differences persisted in driver gene status and the use of targeted therapy. Among patients with fewer than eight BMs (18 in the WBRT group and 46 in the WBRT-SIB group), the WBRT-SIB group demonstrated a significantly improved local PFS compared to the WBRT group (*p* = 0.043) (Figure 4A). Moreover, a trend toward improved intracranial PFS (iPFS) was also observed in the WBRT-SIB group, although it did not reach statistical significance (*p* = 0.066) (Figure 4C). However, no statistically significant differences were observed in distant PFS between the two groups (*p* = 0.775) (Figure 4B).

In the subgroup analysis stratified by targeted therapy status, no significant differences were found in local PFS, distant PFS, or iPFS between the WBRT and WBRT-SIB groups. Similarly, the subgroup analysis stratified by driver gene status and another subgroup analysis based on the sum of the longest diameters of BMs also failed to yield any positive results, with no significant differences observed between the two treatment groups across the three endpoints.

### Treatment-related toxicity

The incidence of treatment-related toxicities was evaluated. Notably, symptomatic radionecrosis occurred in two patients (2.8%) within the WBRT-SIB cohort. One patient presented with seizures 2 months after radiotherapy, and radionecrosis was confirmed on contrast-enhanced MRI; symptoms improved with medical management. The second case was an asymptomatic radionecrosis identified on a routine follow-up MRI 8 months post-treatment. Both patients had received a PTV dose of 30 Gy with a P-GTV boost of 50 Gy. No radionecrosis was observed in the WBRT group.

## Discussion

This single-center retrospective study aimed to evaluate the intracranial efficacy of two radiotherapy modalities—WBRT and WBRT-SIB—in patients with NSCLC-BM, and the analysis revealed no statistically significant differences between the WBRT and WBRT-SIB groups in terms of local PFS, distant PFS, and iPFS before and after PSM analysis. However, a subgroup analysis of patients with fewer than eight BMs demonstrated a significant improvement in local PFS in the WBRT-SIB group, with a trend toward prolonged iPFS that did not reach statistical significance.

In the patient cohort enrolled in our study, a substantial number of individuals with driver gene-positive NSCLC nonetheless received radiotherapy as first-line treatment for their BMs, primarily due to targeted therapy resistance or to alleviate neurological symptoms. Although patients with driver gene-positive NSCLC-BMs can now prioritize targeted therapy, thereby delaying the need for radiotherapy or surgical intervention for intracranial metastases (14), radiotherapy remains a critical treatment modality for those presenting with neurological symptoms at diagnosis, for those with oligoprogressive intracranial lesions following targeted therapy, or for those who are driver gene-negative (15).

The optimal approach to radiotherapy for patients with multiple BMs remains a subject of ongoing debate. According to the *Clinical Practice Guidelines for the Management of Brain Metastases from Non-small Cell Lung Cancer with Actionable Gene Alterations in China (2025 Edition)*, for NSCLC patients with BMs who are in good performance status, have oligometastatic disease (1–3 lesions), and radiologically defined well-circumscribed lesions with intact tumor capsules, SRS or SRT is recommended. However, for patients with more than three metastatic lesions, the optimal radiotherapy approach remains controversial. This debate has been further informed by recent phase III randomized trial data presented at the ASCO 2025 Annual Meeting, which demonstrated that in patients with 5–20 BMs, SRS/SRT resulted in superior patient-reported symptom control and less functional interference compared to hippocampal-avoidance WBRT (HA-WBRT), without compromising overall survival. These findings challenge the traditional paradigm and support the expanding role of SRS/SRT for patients with a higher burden of intracranial disease. In light of this evolving evidence, WBRT at a dose of 30 Gy/10 fractions or 37.5 Gy/15 fractions is generally recommended, but for patients with 5–20 BMs, SRS/SRT may also be considered in well-equipped medical centers.

WBRT-SIB, as an innovative cranial radiotherapy technique, has garnered significant attention due to its ability to deliver low-dose radiation to the entire brain while simultaneously administering a high-dose boost to metastatic lesions. Numerous studies have investigated the efficacy of WBRT-SIB and its impact on patient outcomes. However, most of these studies are retrospective in nature, and their findings remain inconsistent, with no unified conclusion (9, 16–19). In a phase II clinical trial, hippocampal-sparing WBRT-SIB demonstrated comparable intracranial control rates to conventional WBRT combined with SRS (13). Furthermore, a real-world analysis conducted in 2022 revealed that SRS did not result in a statistically significant improvement in survival, iPFS, or distant PFS when compared to WBRT or WBRT-SIB in the management of NSCLC patients (20).

In our study, no statistically significant differences were observed between the two radiotherapy modalities. We hypothesize that this lack of difference may be attributed to the considerable heterogeneity in the number of BMs within our patient population. Notably, the WBRT cohort included 14 patients with more than 10 BMs and 4 patients with over 100 BMs—cases that are typically excluded from other analyses.

In a retrospective study by Zhang et al. (21), data from 82 patients treated with WBRT-SIB and 83 patients who received WBRT were analyzed to evaluate local PFS and iPFS. Their findings indicated that WBRT-SIB was associated with improved local PFS and iPFS, both before and after the application of propensity score matching techniques. However, the number of BMs was not included as a baseline characteristic, nor was its potential influence on treatment outcomes assessed. Similarly, Popp et al. (22) reported that WBRT-SIB, when combined with hippocampal avoidance, led to enhanced local PFS and iPFS. Nevertheless, their patient cohort included up to 17 BMs per individual, and the absence of highly multifocal cases (e.g., > 20 metastases) may not reflect the complexity encountered in real-world clinical settings. Consequently, the generalizability of their findings to patients with extensive intracranial disease remains uncertain, thereby limiting their applicability in broader clinical practice. Furthermore, neither study restricted inclusion by pathological tumor type, nor did they exclude patients who had previously undergone surgical resection or radiotherapy for BMs. In contrast, a 2024 single-arm study demonstrated the efficacy of WBRT-SIB in patients with multiple large and/or diffuse BMs (23), emphasizing the need for further prospective trials to confirm these preliminary findings.

Table 3 provides a summary of recent studies on radiotherapy for BMs. It is evident that the majority did not restrict patients by the pathological subtype of the primary tumor and often included individuals with multiple BMs. Moreover, most studies focused primarily on iPFS as an outcome, while few explored the specific patterns of intracranial recurrence following radiotherapy.

**TABLE 3:** Different researches on brain metastases radiotherapy before our study.

Author	Total sample size	Primary tumor pathology	BM number	Treatment strategy	Conclusion
Wang et al. (16)	112	Not limited	4–15	HA-WBRT-SIB vs. HA-WBRT	HA-WBRT + SIB resulted in better iPFS.
Deng et al. (17)	232	EGFR-mutant lung adenocarcinoma	Not mentioned	WBRT-SIB vs. WBRT vs. local radiotherapy	WBRT-SIB resulted in better iPFS than WBRT while equaled with local radiotherapy.
Ni et al. (18)	684	NSCLC	Not mentioned	WBRT-SIB vs. WBRT vs. SRS	WBRT-SIB resulted in better iPFS.
Ni et al. (19)	263	Small cell lung cancer	Not mentioned	WBRT-SIB vs. WBRT vs. SRS	WBRT-SIB resulted in better iPFS.
Chen et al., (20)	156	NSCLC	1–4	WBRT-SIB vs. WBRT vs. SRS	WBRT-SIB did not result in significantly superior iPFS compared to WBRT or SRS.
Zhang et al. (21)	165	Not limited but conducted a sub-group analysis of NSCLC	Not mentioned	WBRT-SIB vs. WBRT	WBRT-SIB resulted in better local PFS and iPFS.
Popp et al. (22)	124	Not limited	4–17	WBRT-SIB vs. WBRT	WBRT-SIB resulted in better local PFS and iPFS while WBRT achieved better distant PFS.

To mitigate the effects of baseline characteristic imbalances and to generate hypotheses regarding which patient populations might derive the greatest benefit from WBRT-SIB, a subgroup analysis was conducted. In a 2020 phase II clinical trial evaluating hippocampal-sparing WBRT-SIB, patients were stratified into three groups based on the number of BMs: 1–3, 4–6, and 7–8 (13), and the cut-off of eight BMs for our subgroup analysis was selected based on the upper limit used in this trial. The subgroup analysis revealed that among patients with fewer than eight BMs, WBRT-SIB significantly improved local PFS compared to conventional WBRT with a trend toward prolonged iPFS that did not reach statistical significance, thereby supporting our earlier hypothesis. These results suggest that WBRT-SIB may confer substantial tumor control benefits in patients with limited intracranial tumor burden (< 8 BMs), whereas its application in patients with extensive metastatic involvement may be less favorable and therefore not recommended.

Radionecrosis is a well-documented complication of radiotherapy for BMs (24). However, while existing research on radionecrosis has predominantly focused on conventional WBRT and the high-dose-per-fraction regimens used in SRS (25–27), data specifically pertaining to its incidence following WBRT-SIB remain scarce. In the present study, radionecrosis was identified in two patients within the WBRT-SIB cohort, whereas no cases were observed in the WBRT group. It is crucial to note, however, that due to the inherent limitations of our retrospective design such as patient loss to follow-up and the absence of mandatory routine MRI for asymptomatic individuals, the reported incidence of radionecrosis is likely an underestimate. Future prospective studies, incorporating standardized radiographic follow-up, are warranted to definitively establish the incidence and identify the predictive factors for radionecrosis following WBRT-SIB.

The interpretation of our findings must be understood within the context of the specific healthcare environment in which this study was conducted. Although current guidelines increasingly support the use of SRS even in patients with multiple BMs (28), its application in real-world clinical practice is often limited by two key factors: restricted accessibility and socioeconomic barriers. This creates a unique and clinically significant patient population—one that is underrepresented in clinical trials yet frequently encountered in routine practice worldwide. Consequently, our study offers valuable “real-world” evidence regarding the outcomes of these patients.

It is important to note that with the continuous advancements in IMRT, we are now capable of delivering therapeutic doses of WBRT while effectively minimizing radiation exposure to the bilateral hippocampal dentate gyri in patients without metastases near the hippocampi. This approach has been demonstrated to help preserve neurocognitive function, potentially enhancing both treatment tolerability and effectiveness (29, 30). We anticipate that future prospective studies will provide a more comprehensive evaluation of the differences in efficacy and toxicity between HA-WBRT and HA-WBRT-SIB in patients with varying degrees of intracranial tumor burden.

This study’s comparison of WBRT-SIB with WBRT alone reflects, to some extent, the clinical decision-making pathway at our institution for managing multiple BMs. We acknowledge that a comparison with a sequential boost regimen would be more suitable for isolating the specific biological and practical advantages conferred by the “simultaneous” nature of the dose delivery, representing an important direction for future prospective research. Nevertheless, the evidence provided by our study holds clear clinical significance for evaluating the absolute value of the integrated WBRT-SIB strategy relative to conventional palliative WBRT.

This study is subject to several limitations. First of all, due to its retrospective design, randomization in patient allocation was not feasible. As a result, the potential for selection bias between the two treatment groups cannot be excluded, which may have influenced the observed outcomes. Second, key clinical parameters such as overall survival, quality of life, and neurological function were not assessed, primarily due to the lack of comprehensive data. The omission of these critical endpoints restricts a more holistic evaluation of the clinical benefits and trade-offs associated with the two radiotherapy modalities. Third, our patient cohort did not receive neuroprotective interventions, such as hippocampal-sparing radiotherapy or administration of memantine. Future studies are warranted to explore the influence of such neuroprotective measures in the context of WBRT-SIB. Furthermore, this study did not include patients treated with SRT, and its omission means that our findings may not fully reflect the complete spectrum of current radiotherapy practices for NSCLC-BM. Finally, given the relatively small sample size and inherent biases associated with retrospective analyses, the generalizability of our findings is limited. Prospective, large-scale, randomized controlled trials are essential to validate our results and further elucidate the clinical value of WBRT-SIB in patients with varying intracranial disease burdens.

## Conclusion

In summary, no significant differences in local PFS, distant PFS, or iPFS were observed between the WBRT and WBRT-SIB groups among patients with NSCLC-BM, both before and after PSM. However, subgroup analysis revealed that patients with fewer than 8 BMs who received WBRT-SIB experienced a significantly longer local PFS compared to those treated with conventional WBRT (*p* = 0.043), along with a trend toward improved iPFS that did not reach statistical significance (*p* = 0.066). Furthermore, anti-angiogenic therapy showed a trend as a protective factor for local PFS without reaching statistical significance (*p* = 0.086). Collectively, these findings suggest that WBRT-SIB may confer superior local tumor control in selected NSCLC-BM patients with a limited number of BMs (< 8 lesions), while emphasizing the need for further studies to validate these observations in larger, prospective cohorts.

## Data Availability

The raw data supporting the conclusions of this article will be made available by the authors, without undue reservation.

## Footnotes

1
https://brainmetgpa.com/
