# Supplementary material for: Efficacy of whole-brain radiotherapy with or without simultaneous integrated boost in non-small cell lung cancer with brain metastases: a retrospective analysis
Source: Front Med (Lausanne). 2026 Jan 12;12:1733289. doi: 10.3389/fmed.2025.1733289 (PMC12832967; doi:10.3389/fmed.2025.1733289)
Supplement: Supplementary file 1 [file Table_1.docx]

Supplementary Table S1. Baseline Characteristics of Patients with Fewer Than Eight Brain Metastases.

| Characteristics | WBRT | WBRT-SIB | P value |
| --- | --- | --- | --- |
| n | 18 | 46 |  |
| age, median (IQR) | 61 (56, 63) | 59.5 (54, 68) | 0.487 |
| sex, n (%) |  |  | 0.822 |
| Male | 12 (66.7%) | 32 (69.6%) |  |
| Female | 6 (33.3%) | 14 (30.4%) |  |
| GPA, median (IQR) | 2 (1.5, 2.5) | 2 (2, 2.5) | 0.585 |
| Number of BMs, median (IQR) | 3 (2, 4.75) | 3 (1, 4) | 0.276 |
| Sum of maximum diameter of BMs, median (IQR) | 3.6 (2.42, 4.6) | 3.29 (2.2525, 5.09) | 0.782 |
| Sum of volume of BMs, median (IQR) | 18.615 (10.971, 52.661) | 36.564 (5.6153, 63.25) | 0.728 |
| Histological status, n (%) |  |  | 0.346 |
| Large cell carcinoma | 0 (0%) | 3 (6.5%) |  |
| Adenocarcinoma | 18 (100%) | 41 (89.1%) |  |
| Squamous cell | 0 (0%) | 2 (4.4%) |  |
| Driver Gene Status, n (%) |  |  | 0.006 |
| Negative | 2 (11.1%) | 22 (47.8%) |  |
| Positive | 16 (89.9%) | 24 (52.2%) |  |
| Neurologic symptoms, n (%) |  |  | 1.000 |
| Yes | 14 (77.8%) | 35 (76.1%) |  |
| None | 4 (22.2%) | 11 (23.9%) |  |
| Extracranial metastases, n (%) |  |  | 0.303 |
| Yes | 10 (55.6%) | 19 (41.3%) |  |
| None | 8 (44.4%) | 27 (58.7%) |  |
| Chemotherapy, n (%) |  |  | 0.058 |
| Yes | 16 (89.9%) | 30 (65.2%) |  |
| None | 2 (11.1%) | 16 (34.8%) |  |
| Target therapy, n (%) |  |  | 0.002 |
| Yes | 16 (89.9%) | 21 (45.7%) |  |
| None | 2 (11.1%) | 25 (54.3%) |  |
| Anti-angiogenic therapy, n (%) |  |  | 0.807 |
| Yes | 8 (44.4%) | 22 (47.8%) |  |
| None | 10 (55.6%) | 24 (52.2%) |  |
| Immunotherapy, n (%) |  |  | 0.424 |
| Yes | 2 (11.1%) | 11 (23.9%) |  |
| None | 16 (33.3%) | 35 (76.1%) |  |
